# *In Vivo* Efficacy and Biochemical Target Engagement of a Chromene-Based Inhibitor of *Trypanosoma cruzi* Lysyl-tRNA Synthetase

**DOI:** 10.1021/acs.jmedchem.6c00764

**Published:** 2026-08-13

**Authors:** Thaís Cristina Ferreira dos Santos, Caio Cesar de Lima Silva, Amanda Gonçalves Eufrásio, Cristiane Tambascia, Irene Layane De Sousa, Letícia Marchese, Michelle Fagundes Catelli, Leonardo Rodrigues de Almeida, Giovana da Costa Venancio, Valéria Barbosa de Souza, André Almeida Schenka, Silvana Aparecida Rocco, Gustavo Fernando Mercaldi, Artur Torres Cordeiro

**Affiliations:** † Brazilian Biosciences National Laboratory (LNBio), 215006 Brazilian Center for Research in Energy and Materials (CNPEM), Campinas, São Paulo 13083-100, Brazil; ‡ School of Pharmaceutical Sciences, 28132 Universidade Estadual de Campinas (UNICAMP), Campinas, São Paulo 13083-871, Brazil; § Theoretical and Structural Chemistry Group (QTEA), Universidade Estadual de Goiás (UEG), Anápolis, Goiás 75132-400, Brazil; ∥ Department of Pharmacology, School of Medical Sciences, Universidade Estadual de Campinas (UNICAMP), Campinas, São Paulo 13.083-888, Brazil

## Abstract

Aminoacyl-tRNA synthetases (aaRSs) have emerged as essential and attractive antiparasitic targets. In *Trypanosoma cruzi*, *in vivo* evidence supporting lysyl-tRNA synthetase (LysRS) as a valid target has thus far been restricted to a single chemotype. Here, we report the repositioning of a chromene-based LysRS inhibitor developed for *Plasmodium falciparum* and *Cryptosporidium parvum*, providing an independent chemical and structural validation of LysRS in *T. cruzi*. Compound 5 displayed potent activity against intracellular *T. cruzi* amastigotes (EC_50_ = 0.9 μM), low host-cell toxicity, and oral efficacy in an acute murine model of Chagas disease, suppressing parasitemia and reducing tissue parasite burden. Pharmacokinetic (PK) profiling revealed favorable oral bioavailability with delayed absorption, highlighting the importance of PK context in efficacy assessment. Biochemical inhibition and crystallographic structures provide evidence of *T. cruzi* LysRS target engagement. These findings reinforce *T. cruzi* LysRS as a tractable target and reveal a new chemical scaffold for Chagas disease drug development.

## Graphical abstract

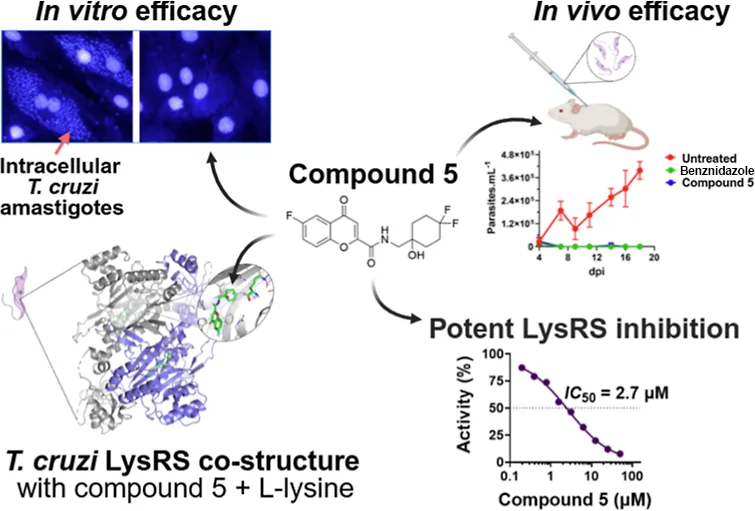

## 1 Introduction

Caused by *Trypanosoma cruzi*, Chagas disease (CD) is endemic in Latin America and has become an emerging concern in nonendemic countries. Recognized as a neglected disease, it presents an acute phase characterized by high levels of parasite replication in tissues, resulting in an elevated parasitemia. If left untreated, it can progress to a chronic form, which is typically asymptomatic but leads to late cardiac complications in approximately 30% of patients.^1,2^ In this context, the search for novel therapeutic targets has become increasingly urgent, primarily because of the significant pharmacological constraints associated with the two available nitroheterocyclic drugs, benznidazole (BNZ) and nifurtimox (NFX). Both drugs present notable adverse effects and limited efficacy, particularly when addressing the chronic phase of disease.^3^

Despite decades of research and recent advances, the translation of promising compounds into clinical trials remains scarce—especially those effective against the chronic phase of the disease—so therapeutic options for Chagas disease continue to be extremely limited and the development of new, effective drugs remains a major challenge.^4,5^ Advances in target identification, validation strategies, and phenotypic screening approaches have expanded the number of hit candidates entering the drug discovery pipeline.^6–9^ These initial hits are then subjected to structural optimization steps aimed at improving their potency, selectivity, and pharmacokinetic profile, resulting in lead compounds that are subsequently evaluated *in vitro* and *in vivo*.^10^

The limitations of current therapies highlight the appeal of targeting essential parasite translation pathways, particularly aminoacyl-tRNA synthetases (aaRSs), which are increasingly recognized as promising therapeutic targets across protozoan pathogens.^11–13^ These enzymes have been validated in diverse pathological contexts, including cancer,^14^ neurodegenerative disorders,^15^ and infectious diseases.^16–18^ Among the aaRSs, lysyl-tRNA synthetase (LysRS) catalyzes the ATP-dependent ligation of l-lysine to the cognate tRNA, a crucial step in protein translation. Recently, Tulloch et al.^19^ reported new quinazolines that target LysRS and are active against *T. cruzi*, *Trypanosoma brucei*, and *Leishmania donovani*. Mutagenesis studies demonstrated that these compounds target the ATP binding site of the enzymes, indicating that LysRS is functionally conserved among trypanosomatids. Additionally, these quinazolines demonstrated moderate efficacy in an *in vivo* murine model of acute Chagas disease, reinforcing the relevance of this target for parasite survival. Despite this progress, it remains unclear whether LysRS represents a broadly tractable antiparasitic target resilient to chemical diversity, as current *in vivo* validation with *T. cruzi* has relied primarily on a single chemotype. This leaves open the question of whether the target’s tractability extends to inhibitors with distinct chemical backbones and, consequently, whether it can support robust antiparasitic drug discovery efforts.

Here, we test whether LysRS inhibition in *T. cruzi* is supported by a chemically distinct scaffold by evaluating compound 5, a chromene derivative previously reported as a potent inhibitor of LysRS in *Plasmodium falciparum* (*Pf*LysRS) and *Cryptosporidium parvum* (*Cp*LysRS).^20^ Compound 5 was optimized by Baragaña and colleagues through a structure-based approach guided by *Pf*LysRS and *Cp*LysRS costructures, had its selectivity over human LysRS (*Hs*LysRS) rationalized through molecular dynamics, and showed oral efficacy in mouse models of malaria and cryptosporidiosis.^20^ In this work, this compound showed potent anti-*T. cruzi* activity *in vitro*, significantly reducing the number of intracellular amastigotes. In an *in vivo* murine model of acute Chagas disease, treatment with compound 5 resulted in a reduction of both parasitemia and tissue parasite burden. Its characterization through biochemical and crystallographic assays further confirmed the action of compound 5 on cytosolic *T. cruzi* LysRS (*Tc*LysRS^cyt^) and its ATP-mimetic nature, reinforcing this enzyme as an attractive target for antichagasic drugs.

## 2 Results

### 2.1 Compound 5 Shows Potent *In Vitro* Activity against *T. cruzi* Amastigotes

To assess the activity of compound 5 (Figure 1A) against amastigote forms of *T. cruzi*, experiments were conducted using an *in vitro* infection model. The compound was tested on H9c2 cardiomyoblasts (ATCC CRL-1446) infected with *T. cruzi* (strain Dm28c) and was found to effectively reduce the number of infected cells, with an EC_50_ of 0.9 μM (Figure 1B). Toxic effects of compound 5 on host cell viability were only observed at higher concentrations (40 μM), indicating selectivity toward the intracellular parasites (Figure 1B). Additionally, samples treated with 12 μM of compound 5 showed a marked reduction in the number of parasites compared to untreated controls (Figure 1C). With a CC_50_ of approximately 40 μM and an *EC*_50_ of 0.9 μM, the resulting selectivity index (SI) exceeds 40, indicating a favorable therapeutic window and supporting the feasibility of progressing to *in vivo* efficacy studies. These results show that a chromene scaffold, chemically distinct from previously reported quinazolines,^19^ retains potent intracellular activity against *T. cruzi*.

**Figure 1:**
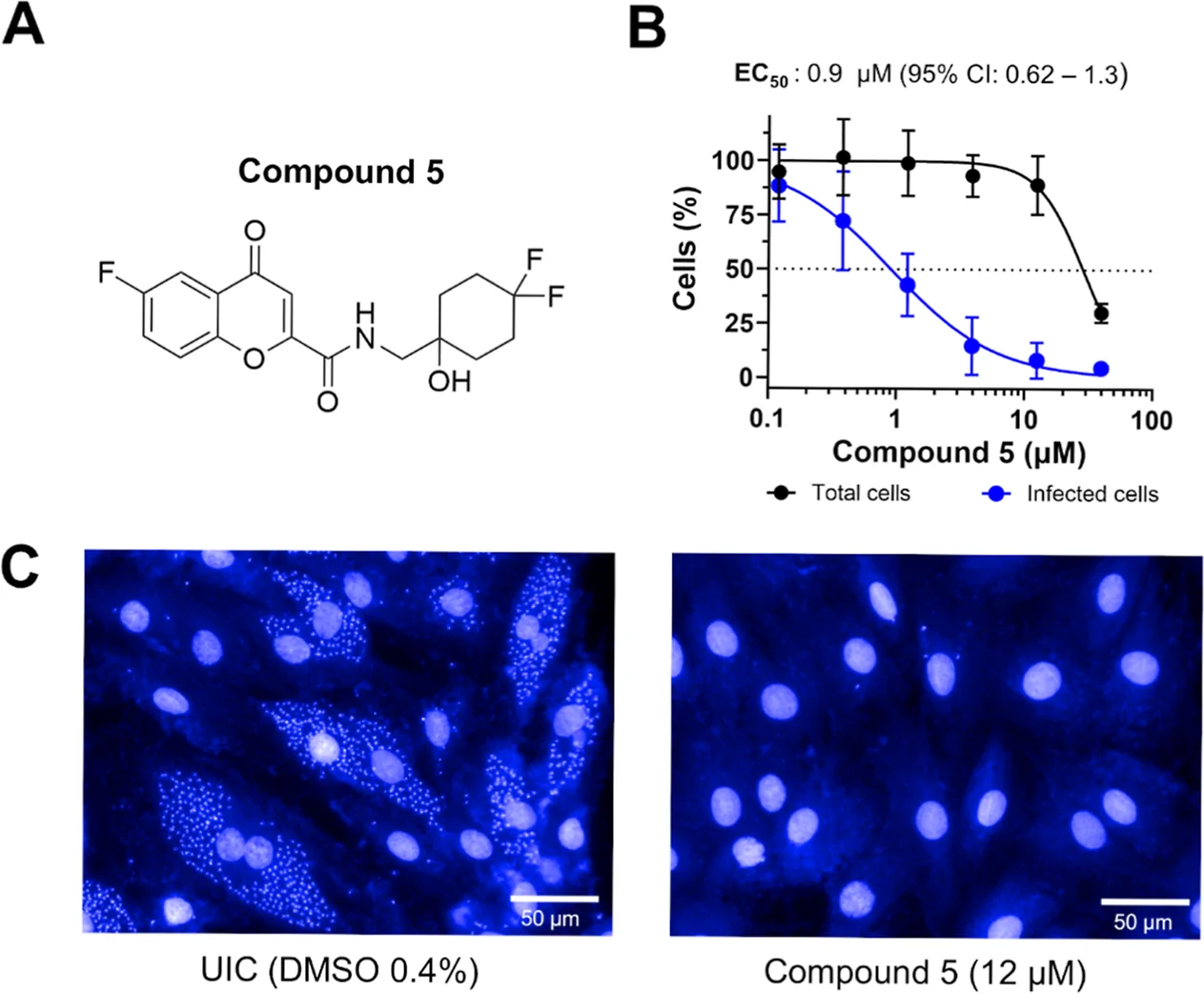
Antiparasitic effects of compound 5 on *Trypanosoma cruzi*. (A) Chemical formula of compound 5. (B) Concentration–response curves illustrating the impact of compound 5 on H9c2 cardiomyoblasts infected with *T. cruzi*. The total counts of host cells (black) and infected cells (blue) were measured after 72 h of exposure to compound 5. (C) Fluorescence microscopy images of H9c2 cells harboring intracellular amastigotes following 72 h of compound 5 treatment. Representative microscopy image of untreated infected cells (UIC, 0.4% dimethyl sulfoxide, DMSO) and compound 5-treated cells (12 μM) is shown. Host cell nuclei and parasite kinetoplasts were stained using Hoechst 33342. Data are presented as means ± standard deviations (SD) from three independent experiments, each with three technical replicates.

### 2.2 *In Vivo* Pharmacokinetic Characterization of Compound 5

To establish the pharmacokinetics (PK) profile of compound 5 in the context of the oral formulation, which differs from those previously reported,^20^ the compound was administered to mice *via* intravenous (IV, *in venam*) and oral routes (PO, *per os*). Following IV administration *via* the retro-orbital plexus at 2 mg·kg^–1^, compound 5 exhibited rapid systemic exposure. The highest plasma concentration (*C*_max_) observed was 838.7 ng·mL^–1^, achieved at 0.08 h (5 min) postdose, reflecting the immediate bioavailability associated with this route of administration (Table 1, Figure 2A). When administered orally (*PO*) at 20 mg·kg^–1^, compound 5 produced a comparable *C*_max_ of 892.5 ng·mL^–1^ (Table 1; Figure 2A). However, the time to reach peak concentration (*T*_max_) was delayed to 6 h, indicating slower absorption *via* the oral route than *via* IV. The terminal elimination half-life (*t*_1/2λ_) was 4.0 h for IV, whereas PO resulted in a longer half-life of 9.0 h. These findings suggest moderate systemic persistence of compound 5, with a prolonged terminal phase observed after oral dosing (Table 1).

**Table 1:** Pharmacokinetic Parameters Following Intravenous and Oral Administration of Compound 5 in Mice

parameter	intravenous (2 mg·kg^–1^)	oral (20 mg·kg^–1^)
*C*_max_ (ng·mL^–1^)	838.7	892.5
*T*_max_ (h)	0.08	6.0
*t*_1/2λ_ (h)	4.0	9.0
AUC_0–8_ (ng h·mL^–1^)	1296.1	-
AUC_0–24_ (ng h·mL^–1^)	-	10,853.4
CL or CL/F (mL·kg^–1^·h^–1^)	1543.0	1842.7
Vd or Vd/F (mL·kg^–1^)	2384.6	22,408.3
F (%)	-	83.7

**Figure 2:**
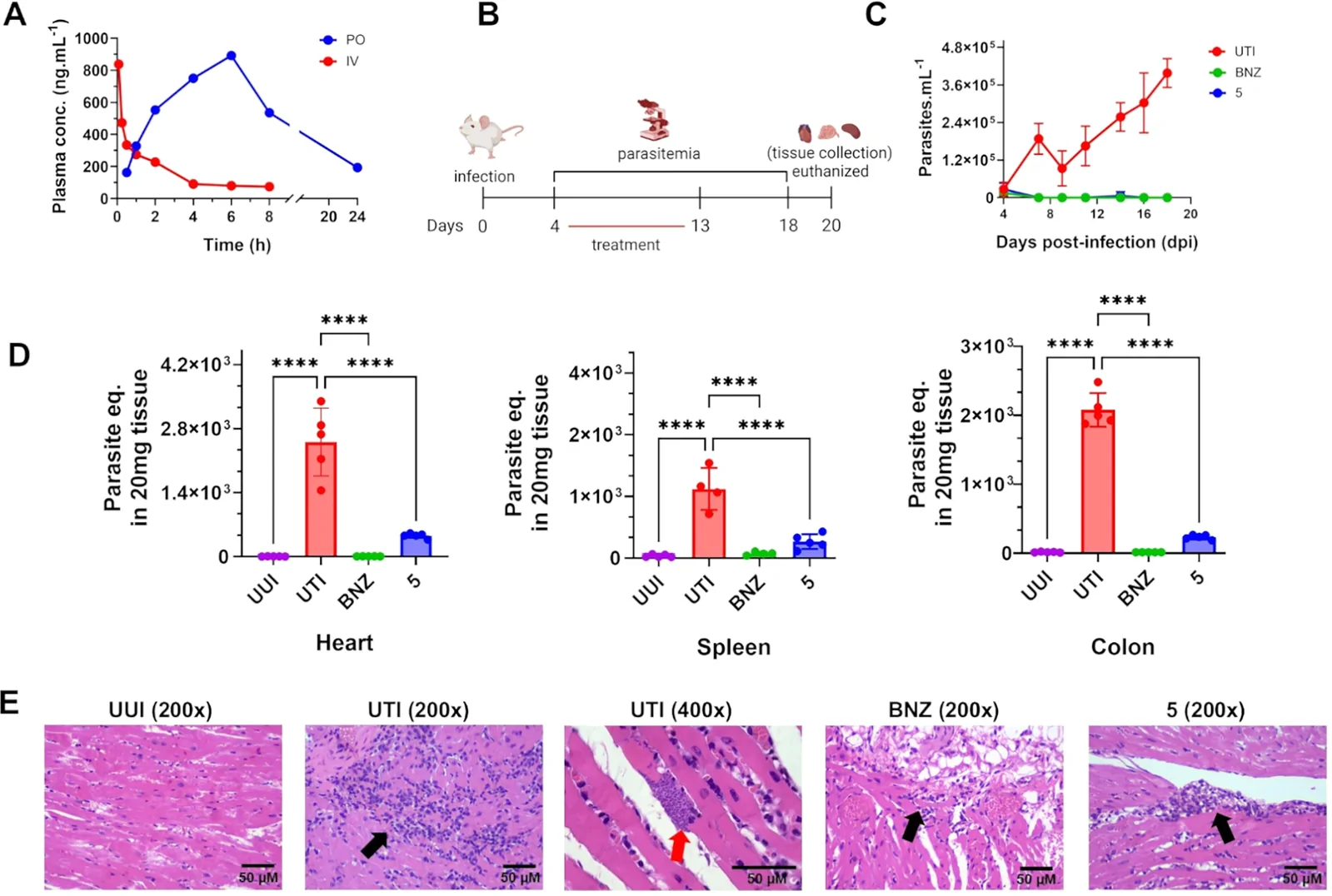
*In vivo* efficacy of compound 5 in a mouse model of acute *Trypanosoma cruzi* infection. (A) Pharmacokinetic profile of compound 5 following administration in BALB/c mice. IV (intravenous administration; *in venam*), PO (oral administration; *per os*). (B) Scheme of experimental design. Female BALB/c mice were infected intraperitoneally with 1 × 10^5^ trypomastigotes of the *T. cruzi* Dm28c strain. (C) Parasitemia curves of animals randomly assigned to groups (*n* = 5): UUI (untreated, uninfected), UTI (untreated, infected), BNZ (infected, treated with benznidazole, 50 mg·kg^–1^, SID, *semel in die*), PO, compound 5 (infected treated with compound 5, 20 mg·kg^–1^ SID, PO). (D) Tissue parasite burden assessed by quantitative real-time PCR (qPCR) in heart, spleen, and colon from infected mice at 20 days postinfection (dpi). Bars represent mean ± standard deviation (SD). Statistical analysis was performed using one-way ANOVA followed by Dunnett’s multiple comparison test. Adjusted *p*-values are indicated by asterisks (****p ≤ 0.0001). (E) Representative hematoxylin and eosin-stained tissue (heart) sections collected after euthanasia. Black arrows indicate inflammatory foci, while red arrows highlight nests of intracellular amastigotes. Images were captured using a bright-field microscope, and the magnifications are indicated in the figure.

Systemic exposure, as reflected by the area under the plasma concentration–time curve (AUC_0–24_ PO and AUC_0–8_ IV), was substantially higher after oral (PO) at 20 mg·kg^–1^ (10,853.4 ng·h·mL^–1^) compared to IV at 2 mg·kg^–1^ (1296.1 ng·h·mL^–1^). Intravenous clearance (CL) was estimated at 1543.0 mL·kg^–1^·h^–1^, while the clearance value determined after oral administration represents the apparent clearance (CL/F), estimated at 1842.7 mL·kg^–1^·h^–1^ (Table 1). Similarly, the volume of distribution following intravenous administration (Vd) was 2384.6 mL·kg^–1^, whereas the substantially higher value estimated after oral dosing corresponds to the apparent volume of distribution (Vd/F), calculated as 22,408.3 mL·kg^–1^ (Table 1). The absolute oral bioavailability (F) was calculated to be 83.7% (Table 1), indicating efficient systemic exposure following oral administration despite the delayed absorption profile (Table 1; Figure 2A). This divergence highlights the sensitivity of aaRS inhibitor exposure profiles to formulation and experimental context. Together, these data define the pharmacokinetic context in which compound 5 was subsequently evaluated for *in vivo* efficacy.

### 2.3 Compound 5 Displays *In Vivo* Efficacy in a Mouse Model of Acute Chagas Disease

In the *in vivo* efficacy assay, compound 5 was administered at 20 mg·kg^–1^, SID (*semel in die*) for 10 days, with a positive control group receiving BNZ at 50 mg·kg^–1^ under the same conditions (Figure 2B). The results showed that compound 5 exhibited efficacy comparable to BNZ as early as the third day of treatment, completely suppressing blood parasitemia in *T. cruzi*-infected mice and maintaining undetectable levels throughout the entire evaluation period (Figure 2C). Notably, this efficacy was achieved using a chemical scaffold and oral formulation distinct from previously reported LysRS inhibitors.^19^

For a more in-depth analysis of the effects of compound 5 treatment on tissue parasite load, quantitative real-time PCR (qPCR) analyses were carried out on heart, spleen, and colon samples collected at the end of the study. In addition, histopathological examinations were performed specifically on heart tissue sections to evaluate the extent of tissue inflammation as well as to identify amastigote nests. qPCR quantification showed that compound 5 treatment significantly reduced parasite load in the heart, spleen, and colon when compared to the untreated infected control (UTI), although values remained slightly higher than those observed with BNZ (Figure 2D).

The histopathological results demonstrate the progression of *T. cruzi* infection across different experimental groups (Figure 2E and Table 2). In cardiac tissue, the UTI exhibits multiple inflammatory foci of varying sizes that are extensively distributed throughout the tissue. The inflammatory infiltrate shows a lymphomononuclear pattern with polymorphonuclear neutrophils (LMNPs). In addition, amastigote nests were detected in all analyzed samples, confirming a high parasite burden. The group infected and treated with compound 5 showed inflammatory foci ranging from rare to scattered with the infiltrate predominantly composed of lymphomononuclear cells (LMN). Despite residual inflammation, no amastigote nests were detected. A similar pattern was observed in the group infected and treated with the BNZ.

**Table 2:** Summary of Cardiac Histopathological Findings^a^

groups	inflammatory foci (IF)	size of IF	type of inflammation	amastigotes nests
UUI	0	–	–	0
UTI	+++	+++	+++	+++
BNZ	+	++	+	0
compound 5	+/++	++/+++	++	0

a Quantification was performed using a semiquantitative scale: inflammatory foci (IF) were classified as absent (0), rare (+, 1–3 foci), sparse (++, 4–6 foci), or multiple (+++, more than 6 foci). The size of the IF was categorized as small (+), medium (++), or large/confluent (+++). The type of inflammation was classified as chronic lymphomononuclear (LMN, +), LMN predominance with occasional neutrophils (++, mixed pattern), or lymphomononuclear with neutrophilic infiltration characterizing active chronic inflammation (LMNP, +++). Amastigote nests were classified as absent (0), rare (+, 1–2 nests), sparse (++, 3 or more nests), or intensely present within the group (+++, detected in all animals). The groups were UUI (untreated, uninfected), UTI (untreated, infected), BNZ (infected, treated with benznidazole), and compound 5 (infected, treated with compound 5).

### 2.4 Kinetics and Structural Studies Reinforce *Tc*LysRS^cyt^ as the Target of Compound 5 in *T. cruzi*

Steady-state kinetic assays were performed to assess the kinetic properties of *Tc*LysRS^cyt^. For this purpose, the full-length recombinant enzyme was produced in *E. coli* and purified by affinity and size exclusion chromatography (Supporting Information appendix, Figure S1A). Analytical gel filtration showed that the purified protein eluted as a predominant nonmonomeric species in solution, consistent with the oligomeric behavior expected for LysRS enzymes (Figure S1B). For the kinetic assays, the enzyme was tested with varying concentrations of ATP or l-lysine (hereafter also referred to as lysine), while the other substrate was kept at a constant, saturating concentration. These assays were conducted in the absence of tRNA, and the initial reaction rates were determined by quantifying AMP formation over time. When lysine was used as the variable substrate, the enzyme displayed typical Michaelis–Menten kinetics, yielding a *K*_M_ value of 96.8 μM and a *V*_max_ of 3.9 pmol·min^–1^ (Figure 3A). In contrast, ATP titration produced a sigmoidal curve, and the data were fitted to the Hill equation, resulting in a *K*_1/2_ of 522.8 μM, a Hill slope (h) of 2.3, and a *V*_max_ of 3.5 pmol·min^–1^ (Figure 3B). Subsequently, inhibition assays using compound 5 demonstrated a dose-dependent reduction in *Tc*LysRS^cyt^ activity, with an IC_50_ of 2.7 μM (Figure 3C), thus confirming the compound’s ability to modulate the function of the *T. cruzi* enzyme.

**Figure 3:**
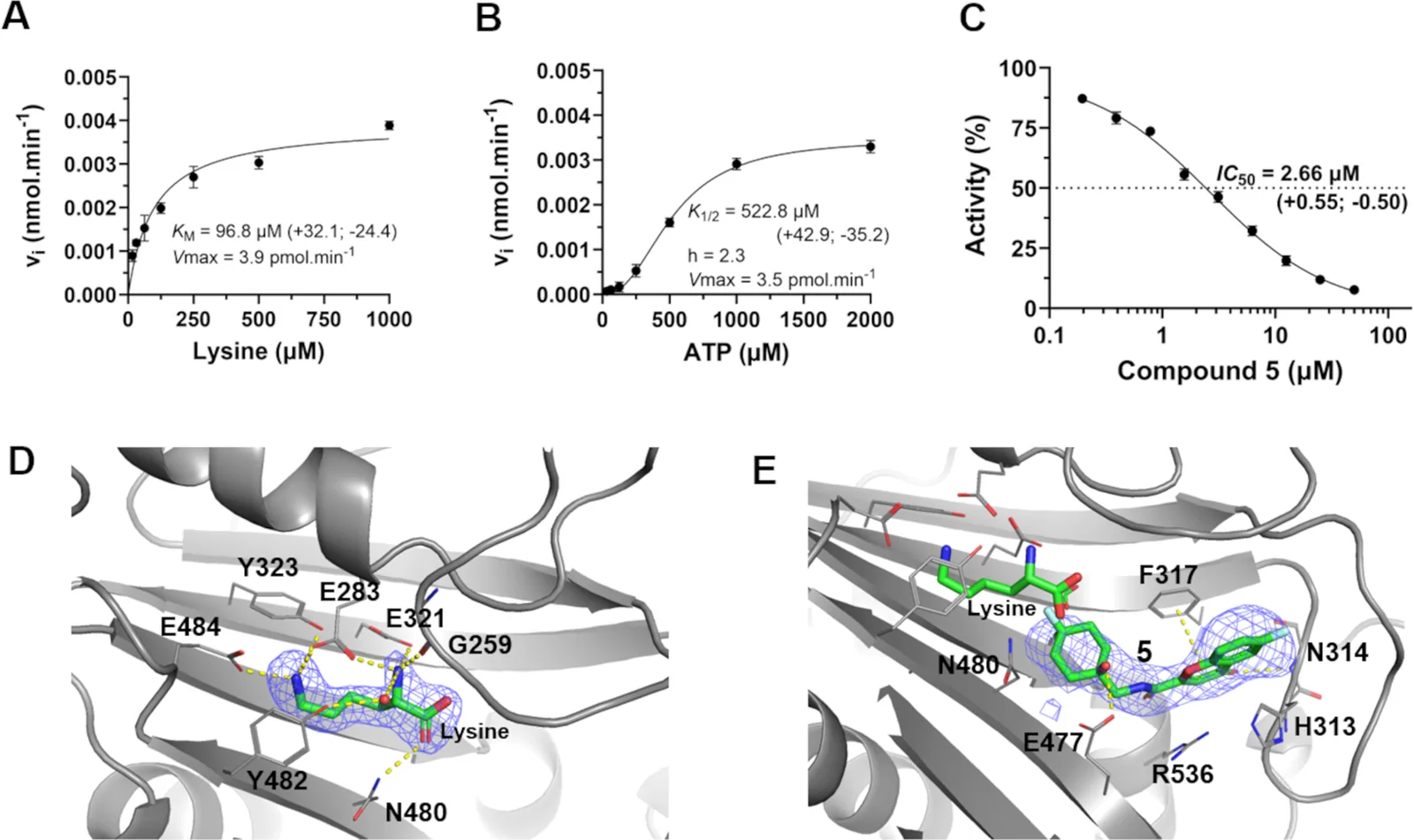
Kinetic properties of *Trypanosoma cruzi* LysRS^cyt^ and validation of enzyme interaction with compound 5. Michaelis–Menten curves obtained for substrates l-lysine (A) and ATP (B), without tRNA in the reaction mixture. The kinetics parameters *K*_M_, *K*_1/2_, h, and *V*_max_ were calculated by nonlinear regression of the data. Concentration–response assay with *Tc*LysRS^cyt^ confirmed the inhibitory effect of compound 5 over the enzyme activity (C). Crystallographic structures *Tc*LysRS^cyt^ depicting the interactions (yellow dash) and Fo-Fc OMIT maps (blue meshes) for l-lysine alone (D) or in combination with compound 5 (E). Ligands are shown as sticks, with carbons colored green. OMIT maps were prepared without ligands in the model and a sigma level of 3 for l-lysine and 2 sigma level for compound 5. Structures were deposited in the protein data bank, under the accession codes 9YJN and 9YJR.

To further validate the interaction and binding mode of compound 5 to *Tc*LysRS^cyt^, differential scanning fluorimetry (DSF) and structural studies were performed. In DSF assays, *Tc*LysRS^cyt^ displayed a biphasic thermal unfolding profile under apo conditions and in the presence of individual ligands. By contrast, incubation with both lysine and compound 5 produced an additional inflection point (Supporting Information appendix, Figure S2). In addition, the first crystal structures of this enzyme were obtained, captured with lysine alone (Figure 3D) and with both lysine and compound 5 (Figure 3E). The asymmetric unit of the crystal contained one LysRS, and the classic dimer could be recovered by generating the symmetry mate (Supporting Information appendix, Table S1 and Figure S3). In the generated models, 57 N-terminal residues and 24 C-terminal residues could not be constructed, indicating that these regions may remain disordered in the refined structures. Compound 5 binds in the ATP pocket of the *Tc*LysRS^cyt^, and its cyclohexyl moiety is situated below lysine, analogous to the binding interactions in LysRS from *Pf*LysRS and *Cp*LysRS^20^ (Figure 4A). Notably, no conformational change in the ATP-binding pocket was observed when comparing the lysine-only complex to the ternary lysine-compound 5 complex.

**Figure 4:**
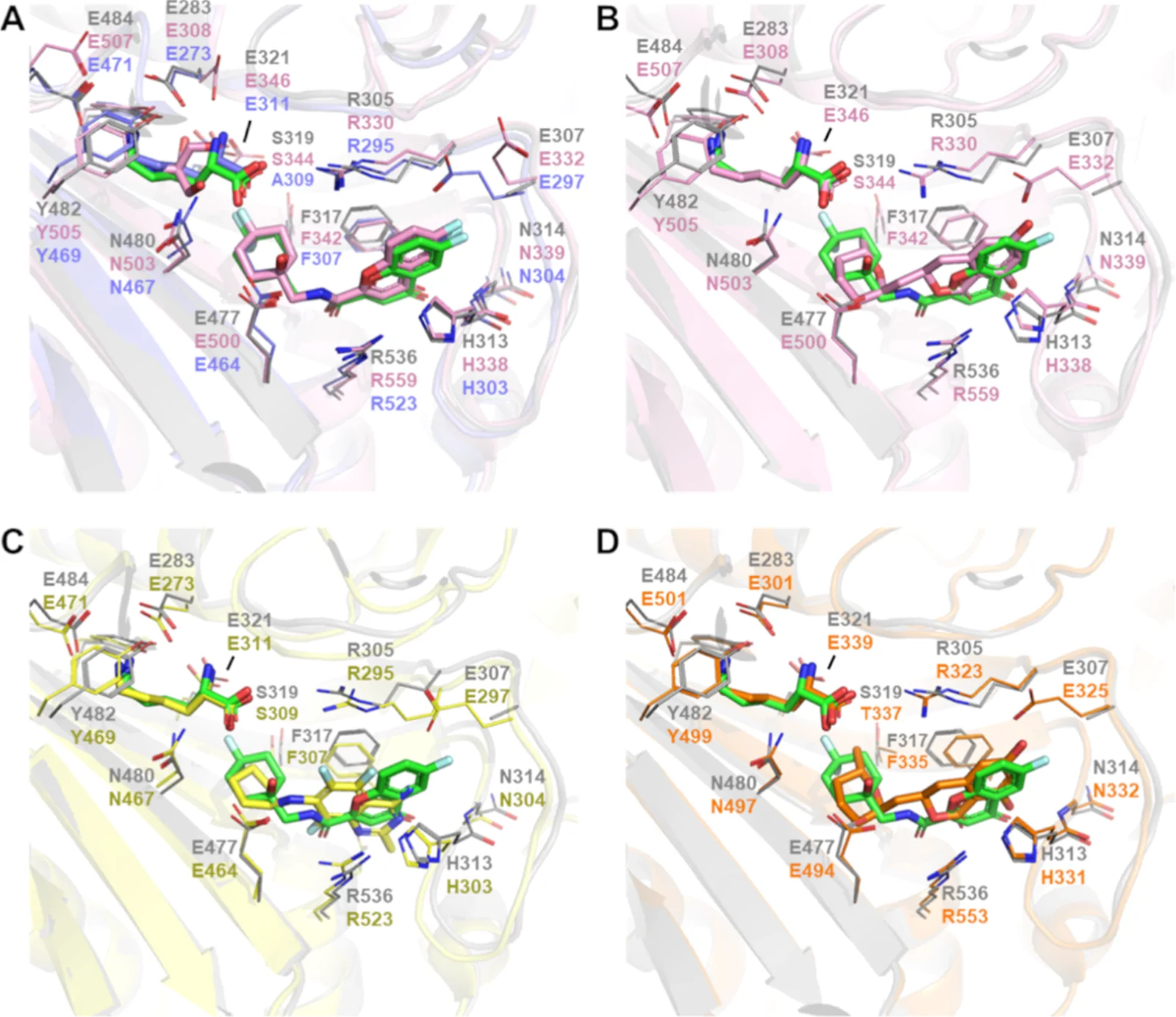
Structural comparison of LysRS complexes with inhibitors. (A) Structural alignment of the LysRS–compound 5–lysine complex from *T. cruzi* (gray with ligands in green; this work) with that from *P. falciparum* (magenta; PDB 6HCU) and *C. parvum* (blue; PDB 6HCW). (B) Comparison of *Tc*LysRS^cyt^–compound 5–lysine (gray with ligands in green, this work) with *Pf*LysRS–cladosporin–lysine complexes (magenta; PDB 4PG3). (C) *Tc*LysRS^cyt^–compound 5–lysine complex (this work) aligned with the *Cp*LysRS–DMU371–lysine complex (yellow; PDB 8S0V). (D) Structural alignment of human (orange; PDB 4YCU) and *T. cruzi* LysRS^cyt^ (gray; this work), in complex with cladosporin (orange) and compound 5 (green) respectively, showing the conservation of the compound 5/ATP binding pockets.

### 2.5 LysRS-Inhibitor Structures Show Conserved ATP-Site Mimicry

The ternary structure of *Tc*LysRS^cyt^ bound to compound 5 and lysine highlights key aspects of inhibitor recognition. In the electron density map, the lysine substrate was well-defined and the cyclohexyl group from compound 5 is positioned in a way that could potentially contribute to the complex stability (Figure 3E). The chromene scaffold is sandwiched between R536/H313 and F317, being engaged in π-stacking with F317. Additionally, the chromene carbonyl forms a hydrogen bond with the main-chain amide of N314, while its cyclohexyl hydroxyl makes a hydrogen bond with E477, anchoring the inhibitor within the ATP-binding pocket (Figure 3E; and sequence alignment in Supporting Information appendix, Figure S4). The ligand positioning and interaction pattern are consistent with the ATP-competitive mechanism proposed for compound 5 by Baragaña et al.^20^ (Figure 4A).

Structural alignments with *Pf*LysRS–cladosporin^21^ (Figure 4B) and *Cp*LysRS–DMU371 ^19^ (Figure 4C) complexes revealed a conserved architecture of the ATP-binding site across kinetoplastid and apicomplexan LysRS enzymes (Figure 4A–C). Pairwise identity between *Tc*LysRS^cyt^ and its orthologs from *P. falciparum*, *C. parvum*, *T. brucei*, *L. donovani*, and *H. sapiens* was 44.1%, 47.7%, 75.0%, 72.8%, and 49.2%, respectively (Supporting Information appendix, Figure S4), with the ATP-binding pocket residues showing a high conservation (80–100%). Compound 5, cladosporin, and DMU371 occupy the adenine subsite and extend toward the ribose-phosphate region, leaving the lysine binding pocket accessible for amino acid substrates—like cladosporin’s mechanism in *P. falciparum*.^22^ Furthermore, a comparison between *Tc*LysRS^cyt^ with human LysRS (*Hs*LysRS) reveals only minor differences in the topology and residue composition of the ATP-binding pocket, primarily due to a polymorphism of S319 in *T. cruzi* corresponding to T337 in the human enzyme (Figure 4D; Supporting Information appendix, Figure S4). Notably, the hydrogen bond network formed by residues Q321, R323, T337, and E339 in *Hs*LysRS—which contributes to the rigidity of the ATP-binding site and compound 5′s selectivity for *Pf*LysRS and *Cp*LysRS^20,22^ —is also conserved in the *Tc*LysRS^cyt^ as Q303, R305, S319, and E321 (Supporting Information appendix, Figure S2; appendix, Figure S4). Together, these structures define conserved ATP-site features and interaction networks that support inhibitor engagement without inducing major conformational rearrangements.

## 3 Discussion

Drug repurposing is a promising strategy for identifying new therapeutic alternatives, particularly for Chagas disease and the limitations associated with BNZ.^5^ This study was designed to move beyond compound-centric validation and instead evaluate whether lysyl-tRNA synthetase represents a chemically and structurally robust target suitable for repositioning across divergent parasitic lineages. In this context, we focused our attention on small molecules that target aminoacyl-tRNA synthetases (aaRS), with a particular focus on LysRS, considering that this is a validated target in *T. cruzi*.^19^ Presently, only 3 other studies have explored aaRS as drug targets in *T. cruzi*, including histidyl-tRNA synthetase,^23^ alanyl-tRNA synthetase,^24^ and methionyl-tRNA synthetase.^25^ Distinct from Tulloch et al.,^19^ we focus our efforts on investigating compound 5, described as a selective inhibitor of *Pf*LysRS and *Cp*LysRS, as a candidate for repositioning against *T. cruzi*.

The *in vitro* activity of compound 5 against intracellular *T. cruzi* amastigotes (strain Dm28c) revealed greater potency than BNZ, with an *EC*_50_ of 0.9 μM compared to 2.2 μM for BNZ.^26^ Cytotoxicity in the host cell was observed at concentrations above 37 μM, with a selectivity index (SI) of approximately 40. Regarding parasites, an SI ≥ 10 is generally considered acceptable to rule out relevant cytotoxicity at the concentrations used in cell-based assays, whereas values above 20 are regarded as adequate and indicative of good selectivity.^27^ This supports a favorable therapeutic window, particularly for *in vivo* studies, and provides a safe margin for dose adjustments. While compound 5 is less potent *in vitro* than the benchmark quinazoline DMU759 (EC_50_ = 0.1 μM for the Sylvio X10/7A1 and Tulahuen strains),^19^ its favorable selectivity index and robust *in vivo* exposure differentiate it as a chemically distinct and viable starting point for optimization.

Beyond *T. cruzi*, the activity profile of compound 5 aligns with its performance in other protozoan parasites, showing inhibition of *P. falciparum* (EC_50_ = 0.27 μM) and *C. parvum* (EC_50_ = 1.3 μM),^20^ as well as *T. brucei* (EC_50_ = 0.6 ± 0.01 μM) and *L. donovani* (EC_50_ = 0.7 ± 0.01 μM).^19^ Cladosporin, another LysRS inhibitor, displayed an EC_50_ of 1.1 μM and 45–90 nM against *L. donovani* and *Plasmodium* strains, respectively.^28,29^ Together, these data support the conclusion that LysRS is a biologically essential and pharmacologically tractable enzyme across diverse protozoan pathogens.

In this work, the pharmacokinetics profile of compound 5 was re-evaluated to check for impacts related to formulation employed. In a previous study, compound 5 displayed low systemic clearance following intravenous administration (3 mg·kg^–1^, IV; CL = 3.4 mL·min^–1^·kg^–1^ = 204 mL·kg^–1^·h^–1^), with a moderate terminal half-life (*t*_1/2λ_ = 2.5 h) and a steady-state volume of distribution (*V*_d_) of 1000 mL·kg^–1^.^20^ In our study, we observed a higher clearance following IV (2 mg·kg^–1^; CL = 1543.0 mL·kg^–1^·h^–1^), a larger apparent volume of distribution (*V*_d_ = 2384.6 mL·kg^–1^), and a slightly longer terminal half-life (*t*_1/2λ_ = 4.0 h).

After oral administration, compound 5 was reported to be rapidly absorbed (*T*_max_ = 2 h), reaching a maximum observed concentrations (*C*_max_) 5.4 μg·mL^–1^ and complete oral bioavailability (*F* = 100%) at 10 mg·kg^–1^.^20^ In comparison, we observed a slower absorption profile (*T*_max_ = 6 h) and a lower maximum concentration (*C*_max_ = 0.9 μg·mL^–1^), despite using a higher oral dose (20 mg·kg^–1^). Nevertheless, the compound maintained a high absolute oral bioavailability (*F* = 83.7%). Taken together, the substantially delayed *T*_max_ observed for compound 5 suggests slower absorption relative to previous reported data, without significantly affecting bioavailability (*F*) that indicates efficient overall systemic uptake despite the reduced absorption rate.^20^

The differences observed between the pharmacokinetic profiles may be related to the distinct experimental conditions used in the two studies, particularly the oral formulation and analytical matrix. Baragaña et al.^20^ used a formulation composed of hydroxypropyl methylcellulose (HPMC), Tween 80, and benzyl alcohol, whereas the present study employed dimethyl sulfoxide (DMSO), Tween 80, polyethylene glycol 400 (PEG400), and phosphate-buffered saline. Such formulation composition differences can influence compound solubility, gastrointestinal stability, dissolution rate, and systemic absorption.^30^ This is consistent with the delayed *T*_max_ observed in this study (6 h versus 2 h reported previously) and a lower *C*_max_ compared with previously reported values.^20^ In addition, compound concentrations were quantified in whole blood in the previous study^20^ versus plasma in the present work, a difference that may affect the derived pharmacokinetic parameters. Thus, while compound 5 retained high oral bioavailability in our model, these comparisons highlight the importance of reassessing PK under the specific formulation, matrix, and efficacy condition used for *in vivo* studies.

In our *in vivo* efficacy model, compound 5 exhibited antiparasitic activity comparable to that of BNZ, maintaining undetectable parasitemia levels throughout the entire evaluation period, starting as early as the third day of treatment. Regarding LysRS inhibitors, our findings align with those reported by Tulloch et al.,^19^ who evaluated the quinazoline *Tc*LysRS inhibitor DMU759 against the *T. cruzi* CL-Brener strain. In their study, DMU759 reduced the parasite load by 81%, with pharmacokinetics identified as the main limitation to achieving a sterile cure. Consistent with this, our tissue parasite burden analysis showed that compound 5 reduced parasite load by 82.4% in the heart, spleen, and colon compared to the untreated (UTI) group as measured by qPCR. Histopathological evaluation revealed that the UTI group exhibited predominantly LMNP infiltrates and amastigote nests, characteristic of active Chagas’ myocarditis in the acute phase of the disease,^31^ with the presence of neutrophils indicating ongoing tissue damage and parasite-driven inflammation.^32^ In contrast, animals treated with compound 5 showed an LMN infiltrate pattern and absence of amastigote nests, indicative of a shift toward nonactive chronic inflammation, a result also seen in the BNZ-treated animals. Together, these findings emphasize that pharmacokinetic contextualization is not ancillary but central to target validation when repositioning aaRS inhibitors across disease models.

Structural analysis of *Tc*LysRS^cyt^, both with lysine alone and in combination with compound 5, shows that the residues forming the ATP-binding pocket maintain their conformations regardless of inhibitor binding. This suggests that, in the trypanosomal enzyme, the rigidity of the ATP-binding pocket may be due to lysine binding—consistent with the typical ordered sequential mechanism of LysRS enzymes, where lysine binds first, followed by ATP^33^—and a network of interactions involving residues Q303, R305, S319, and E321, which closely mirror the network described for the human enzyme.^20^ This structural rigidity helps explain the inhibition profile observed for *Tc*LysRS^cyt^, where compound 5 demonstrates similar potency to that seen in *Hs*LysRS. Our structural data support a conserved ATP-pocket mimicry mechanism analogous to that reported for inhibitors of apicomplexan LysRS enzymes, reinforcing LysRS as a cross-phylum druggable target while open opportunities to exploit regions adjacent to the ATP pocket for selectivity.

Beyond biochemical and structural evidence, on-target engagement of *Tc*LysRS^cyt^ by compound 5 is further supported by published genetic validation in *T. cruzi*. Tulloch et al.^19^ reported that introduction of the S319L mutation in the cellular *Tc*LysRS^cyt^ gene of *T. cruzi* epimastigotes increases the cellular EC_50_ against compound 5 by approximately 25-fold, with comparable resistance shifts observed in *T. brucei* and *L. donovani* bearing the equivalent mutations. This residue corresponds to a conserved serine within the ATP-binding pocket (Supporting Information, Figure S4), previously shown to confer resistance to compound 5 in *P. falciparum* when mutated to leucine (S344L).^20^ This single-residue genetic evidence, together with our biochemical inhibition (IC_50_ = 2.7 μM), DSF stabilization, and crystallographic visualization of compound 5 in the ATP-binding pocket of *Tc*LysRS^cyt^, makes a dominant off-target contribution unlikely. The modest ∼3-fold difference between biochemical IC_50_ and intracellular EC_50_ against amastigotes (0.9 μM) is consistent with intracellular compound accumulation and differences in assay context (notably the absence of tRNA and the recombinant enzyme environment) rather than with a major secondary target. Given the high conservation of the ATP-binding pocket among kinetoplastid LysRS enzymes (>80% residue identity with *Tc*LysRS^cyt^; Supporting Information appendix, Figure S4), the documented *in vitro* activity of compound 5 against *T. brucei* and *L. donovani*,^19^ as well as the convergent resistance phenotype conferred by equivalent S → L mutations across the three parasites,^19^ it is reasonable to anticipate that compound 5 and chemically related chromene scaffolds will retain on-target activity across kinetoplastid pathogens, positioning cytosolic LysRS as a promising pan-trypanosomatid target.

## 4 Conclusions

In summary, this work reinforces LysRS as a valid therapeutic target for acute Chagas disease and demonstrates that its tractability extends beyond the quinazoline chemotype previously described.^19^ Compound 5 displayed potent anti-*T. cruzi* activity *in vitro* and *in vivo*, favorable oral exposure, and evidence of target engagement supported by biochemical inhibition, DSF assays, crystallographic structures, and published genetic validation. In addition, the data indicate that pharmacokinetic contextualization is central to target validation when repositioning compounds across the disease models. Notably, compound 5 represents a distinct chemical class of LysRS inhibitors, offering new avenues for anti-*T. cruzi* drug discovery. Furthermore, we report the first crystallographic structures of *Tc*LysRS^cyt^, alongside a comprehensive kinetic characterization using a fluorescence-based assay that is compatible with high-throughput screening. Overall, this work provides a structural and biochemical framework that facilitates future structure-based drug discovery initiatives for Chagas’ disease. Given the high conservation of the ATP-binding pocket across kinetoplastid LysRS enzymes and previously reported resistance data across multiple parasites, our findings suggest that chromene-based LysRS inhibitors may also support drug discovery efforts against human African trypanosomiasis and leishmaniasis.

## 5 Material and Methods

### 5.1 Compounds and Reagents

ATP, AMP, NAD^+^, lysine, resazurin, salts, DMSO, DMEM, DPBS, and paraformaldehyde were obtained from Merck-Sigma-Aldrich (St. Louis, MO). *Clostridium kluyveri* diaphorase was acquired from Worthington Biochemical Corporation (Lakewood, NJ, USA). Hoechst 33342 was obtained from Thermo Fisher Scientific (Waltham, MA, USA). The compound used in this study, referred to as compound 5,^20^ was obtained from MedChemExpress (Cat. No. HY-126130, Monmouth Junction, NJ, USA). This compound is also known as DDD01510706. According to the supplier, the compound was provided with >98% purity and was stored according to the manufacturer’s recommendations until use.

### 5.2 Parasite Inhibition Assay

For the intracellular amastigote assay, the compound was dissolved and serially diluted in DMSO using a half-log dilution across six concentrations by an ECHO 650 acoustic dispenser (Beckman). Intermediate plates were prepared by diluting compounds 1:25 in DMEM supplemented with 10% FBS. Trypomastigotes (from LLC-MK2 cultures, 6 days postinfection) were used to infect H9c2 rat cardiomyoblasts overnight (16–18 h) at MOI 1. After infection, cells were washed with PBS, trypsinized, and resuspended in DMEM at 2.3 × 10^4^ cells·mL^–1^. Then, 65 μL per well was plated in 384-well plates and incubated for 24 h at 37 °C with 5% CO_2_ and >95% humidity. Subsequently, 7.5 μL of the compound solution was added (final DMSO concentration of 0.4%). After 72 h of treatment, cells were fixed with 4% paraformaldehyde and stained with Hoechst 33342 (4 μg·mL^–1^). Images were acquired using the Operetta High-Content Imaging System (PerkinElmer), six fields per well, and analyzed with Columbus software to quantify the total number of cells and infected cells (≥3 parasite spots). The experiment was performed in biological triplicate, and data were analyzed with GraphPad Prism v.10. (Boston, MA, USA). Results are presented as mean ± SD. EC50 values were determined by nonlinear regression of normalized data using a variable-slope sigmoidal model.

### 5.3 Pharmacokinetic Profiling

Compound 5 was administered as a bolus intravenous solution at a dose of 2 mg·kg^−1^ (dose vehicle: 10% DMSO, 40% PEG400, and 50% Milli-Q water) to female Balb/c mice (*n* = 6) or administered orally by gavage as a solution at a dose of 20 mg·kg^−1^ (dose vehicle: 2% DMSO, 5% Tween 80, 43% PEG400, and 50% PBS) to female Balb/c mice (*n* = 6). The animals, supplied by Multidisciplinary Center for Biological Research (CEMIB) at University of Campinas (Campinas, SP, Brazil), were acclimated for at least 7 days in the Animal Handling and Experimentation Laboratory (LMEA), at Brazilian Biosciences National Laboratory (LNBIO) in Brazilian Center for Research in Energy and Materials (CNPEM) (Campinas, SP, Brazil) before the start of the experiment. They were maintained under standard conditions, with a 12 h light–dark cycle, controlled temperature (22–24 °C), and free access to food and water (ad libitum). Blood samples were collected at 5, 15, and 30 min, and 1, 2 (*n* = 3), 4, 6, 8, and 24 h postdose (*n* = 3) using submandibular vein puncture, retro-orbital plexus, and intracardiac (terminal) puncture. The mice were anesthetized by inhalation of 3% isoflurane at the time of sampling to ensure animal welfare. Blood samples were immediately transferred to tubes containing heparin and processed by centrifugation (4000 g, 10 min, 4 °C) to separate the plasma. The obtained plasma was transferred to clean vials and stored at −80 °C until analysis.

Extraction of compound 5 from mouse plasma was performed using 5 μL of plasma for calibration standards and study samples. Protein precipitation was carried out by adding an extraction solvent composed of acetonitrile and water (50:50, v/v) containing 0.1% formic acid, resulting in a final extraction volume of 100 μL. After solvent addition, samples were mixed thoroughly and then centrifuged at 18,000 *g* for 5 min. The resulting supernatant was analyzed by LC–MS/MS under the conditions described below. The extraction recovery of compound 5 was determined by comparing the peak area responses obtained from plasma samples spiked with the analyte prior to extraction with those from blank plasma extracts spiked with equivalent concentrations of compound 5 after extraction. The mean recovery across the evaluated concentration range was 97%, indicating efficient and reproducible extraction from the plasma matrix.

Chromatographic separation was performed using an ACQUITY UPLC system (Waters, USA, MA, Milford) equipped with a binary solvent manager and sample manager. The analyte was separated on an ACQUITY UPLC BEH C18 column (2.1 × 10 mm, 1.7 μm particle size), protected by a BEH C18 guard column, and maintained at 40 °C. The mobile phase consisted of water containing 0.1% formic acid (v/v) (solvent A) and acetonitrile containing 0.1% formic acid (v/v) (solvent B). The flow rate was set to 0.45 mL·min^–1^, and the total run time was 5.0 min. The gradient elution program was as follows: initial conditions of 75% A/25% B, held for 1.0 min, followed by a linear gradient to 5% A/95% B over 3.0 min, which was maintained until 4.0 min. The system was then returned to the initial conditions (75% A/25% B) at 4.5 min and re-equilibrated until 5.0 min. Compound 5 had a mean retention time of 2.33 min. The autosampler temperature was maintained at 25 °C. The injection volume was 1 μL. Needle washing was performed using acetonitrile/water (50:50, v/v), with pre- and postinjection wash times of 6 s each to minimize carryover.

Detection was carried out using a Xevo TQ-XS triple quadrupole mass spectrometer (Waters, Milford, MA, USA) equipped with an electrospray ionization (ESI) source operating in positive ion mode. Quantification of compound 5 was performed using multiple reaction monitoring (MRM). The protonated molecular ion of compound 5 was monitored at *m*/*z* 355.7 → 108.5 (quantifier transition) and *m*/*z* 355.7 → 164.9 (qualifier transition). The cone voltage was set to 34 V, and collision energies were optimized at 32 and 34 eV, respectively. The dwell time for each transition was 0.025 s. The chromatographic retention window for compound 5 was set between 2.2 and 2.7 min. Data acquisition and processing were performed using MassLynx software (Waters).

Quantification of compound 5 in mouse plasma was performed by using an external calibration curve prepared by spiking blank plasma with known concentrations of the analyte. Calibration standards were prepared at 3.125, 6.25, 12.50, 25.00, 50.00, 100.00, and 200.00 nM, covering the concentration range expected for pharmacokinetic evaluation. Each calibration level was processed by using the same sample preparation procedure applied to study samples. The calibration curve was generated by plotting the peak area response versus the nominal concentration and was fitted by linear regression. The resulting regression equation was *y* = 210.61*x* + 51.433, with a coefficient of determination of *R* ^2^ = 0.9938. The retention time of compound 5 was consistent across all calibration levels and samples, confirming the chromatographic stability and reproducibility throughout the analytical run.

This study was approved by the CNPEM Animal Ethics Committee (CEUA-CNPEM) under protocol number 114, conducted in accordance with the ARRIVE (Animal Research: Reporting *In Vivo* Experiments). Reporting of *in vivo* experiments guidelines as described at https://arriveguidelines.org.

### 5.4 Analysis of Pharmacokinetic Data

Pharmacokinetic parameters were determined by noncompartmental analysis (NCA) according to standard pharmacokinetic principles described by Gabrielsson and Weiner.^34^ The maximum observed plasma concentration (*C*_max_) and the time to reach *C*_max_ (*T*_max_) were obtained directly from the experimental concentration–time data. The terminal elimination rate constant (K_el_) was estimated by log–linear regression of the terminal phase of the plasma concentration–time curve, and the elimination half-life (*t*_1/2 λ_) was calculated as ln(2)/K_el_. The area under the plasma concentration–time curve from time zero to the last measurable concentration (AUC_0–t_) was calculated using the linear trapezoidal method. Apparent oral clearance (CL/F) was calculated as Dose/AUC_0–t_, and the apparent volume of distribution (*V*_d_/*F*) was determined as (CL/F)/K_el_. Pharmacokinetic parameters were calculated separately for intravenous and oral administration.

### 5.5 *In Vivo* Murine Model of Acute *T. cruzi* Infection

For the efficacy assays, female Balb/c mice aged 8–9 weeks (5 animals per group) were used, and the animals were acclimated as previously described. The mice were infected *via* intraperitoneal (IP) injection with 10^5^ bloodstream trypomastigotes of the *T. cruzi* Dm28c strain. The experimental compound 5 was prepared as a suspension containing 2% DMSO, 5% Tween 80, 43% PEG400, and 50% PBS and administered by oral gavage at 20 mg·kg^–1^ SID, starting 4 days postinfection, for 10 days. A negative control (vehicle) and a positive control with BNZ (50 mg·kg^–1^, SID) were used under the same conditions. This study was approved by the CNPEM Animal Ethics Committee (CEUA-CNPEM) under protocol number 115 and conducted in accordance with the ARRIVE (Animal research: Reporting of *in vivo* Experiments) guidelines as described at: https://arriveguidelines.org. Data were analyzed using GraphPad Prism v.10 using one-way ANOVA followed by Dunnett’s multiple-comparison test. Statistical significance was defined as *P* ≤ 0.05.

### 5.6 Parasitemia and Tissue Parasite Burden Quantification

Parasitemia was quantified three times per week by collecting fresh blood samples (5 μL) from the tail vein of mice. The number of circulating parasites was estimated using a Neubauer chamber following the Pizzi-Brenner method. For tissue parasite burden, DNA was extracted from 20 mg of heart, spleen, and colon samples using the commercial Wizard Genomic DNA Purification Kit (Promega Corporation, Madison, WI, USA) according to the manufacturer’s instructions. The qPCR was performed using SYBR Green PCR Master Mix (Applied Biosystems, Foster City, CA, USA) with 75 ng of template DNA and 500 nM of *T. cruzi*-specific primers: TCZ-F (5′-GCTCTTGCCCACAAGGGTGC-3′) and TCZ-R (5′-CCAAGCAGCGGATAGTTCAGG-3′). Standard curves for parasite load quantification were prepared for each tissue type by spiking 1 × 10^6^ *T. cruzi* epimastigotes into 20 mg of noninfected mouse tissue, followed by DNA extraction (as mentioned above) and a 10-fold serial dilution in Milli-Q water.

### 5.7 Histopathological Analyses

Animal hearts were fixed in 10% formalin for 24 h, followed by dehydration in a graded ethanol series (70% to 99.8%). Samples were then cleared in xylene and embedded in paraffin suitable for histological analysis. Sections of 4 μm thickness were obtained using a rotary microtome (Leica Multicut, Wetzlar, Germany), stained with hematoxylin and eosin, and mounted on slides with Entellan (Merck, Darmstadt, Germany). The tissues were examined under a bright-field photomicroscope (Axio Scope A1, Carl Zeiss, Oberkochen, Germany), and digital images were acquired. Histopathological evaluation was conducted using a semiquantitative approach, in which typical alterations in the examined organ were identified and characterized.

### 5.8 Gene Cloning, Protein Expression, and Purification

The full-length coding gene of cytosolic Lysyl-tRNA synthetase from *T. cruzi* (*Tc*LysRS^cyt^), annotated in TriTrypDB Web site with 580 amino acid residues accession number C4B63_45g239,^19,35^ was amplified by PCR from the genomic DNA of *T. cruzi* strain Dm28c. The oligonucleotides used for amplification were TcLysRS1-For (5′-CAGTCAGGATCCATGTCATCCACGAATGAGAC-3′) and TcLysRS1-Rev (5′-TGACTGAAGCTTTTAGAGGAGAGGTACGCCCT-3′). The PCR product was subsequently cloned into the pET28a-SUMO expression vector using *Bam*HI and *Hin*dIII restriction enzymes (New England Biolabs, Ipswich, MA, USA). The resulting plasmid was initially transformed into *E. coli* Mach-1 and chemically competent cells. Gene cloning was verified by PCR and Sanger sequencing.

*Tc*LysRS^cyt^ was produced in *E. coli* BL21­(DE3) cells that had been transformed with the expression plasmid of interest. For protein production, bacterial cultures were grown in Luria–Bertani (LB) medium supplemented with kanamycin (50 μg·mL^–1^). Cultures were incubated at 37 °C with shaking at 200 rpm until they reached an optical density at 600 nm of 0.6–0.8. Protein expression was induced by the addition of 0.5 mM isopropyl β-D-1-thiogalactopyranoside (IPTG). Following induction, the temperature was lowered to 20 °C, and incubation continued for 18 h. Cells were harvested by centrifugation and subsequently resuspended in buffer A (50 mM Tris–HCl pH 8; 500 mM NaCl; 5% glycerol). Cells were lysed using lysozyme (0.1 mg·mL^–1^ of liquid culture) and sonication, followed by centrifugation 40,000 *g* for 1 h to separate the soluble protein supernatant from the cell debris. *Tc*LysRS^cyt^ purification was performed using an ÄKTA system (Cytiva, Uppsala, Sweden) and immobilized metal affinity chromatography (IMAC) on a 5 mL HisTrap HP column (Cytiva). The elution was performed in buffer A containing 250 mM the imidazole (buffer B). Subsequently, the 6xHis and SUMO tags were cleaved using SUMO-protease ULP-1. The cleaved tags were then separated by reverse IMAC on a 5 mL HisTrap HP column (Cytiva). The last purification step involved a size exclusion chromatography, performed using a HiLoad 16/600 Superdex 200 column (Cytiva) in buffer C (50 mM Tris–HCl pH 8; 300 mM NaCl).

Enzymes necessary for the enzymatic coupled assays, including *Saccharomyces cerevisiae* Adenosine 5′-monophosphate deaminase (ScAMD1, E.C. 3.5.4.6) and *Campylobacter jejuni* Inosine-5′-monophosphate dehydrogenase (CjIMPDH, E.C. 1.1.1.205), were expressed and purified as previously described.^36^

### 5.9 Oligomeric State Analysis by Analytical Size-Exclusion Chromatography

The oligomeric state of purified full-length *Tc*LysRS^cyt^ was analyzed by analytical size-exclusion chromatography using a Superdex 200 10/30 column equilibrated with 50 mM Tris–HCl, pH 8.0, and 300 mM NaCl. Chromatographic runs were performed at a flow rate of 0.5 mL·min^–1^, and protein elution was monitored by absorbance at 280 nm. The column was calibrated using selected standards from the Gel Filtration Molecular Weight Markers Kit (Sigma-Aldrich, MWGF), namely, ferritin (440 kDa, 0.3 mg·mL^–1^), β-amylase (200 kDa, 4.0 mg·mL^–1^), alcohol dehydrogenase (150 kDa, 5.0 mg·mL^–1^), albumin (66 kDa, 10.0 mg·mL^–1^), and carbonic anhydrase (29 kDa, 3.0 mg·mL^–1^). Blue dextran (1.0 mg·mL^–1^) was analyzed separately and used to determine the column void volume (V0). Standards and purified *Tc*LysRS^cyt^ (7 mg·mL^–1^) were injected at 100 μL in independent runs, including ferritin alone, β-amylase combined with alcohol dehydrogenase, albumin combined with carbonic anhydrase, and *Tc*LysRS^cyt^. A calibration curve was generated by plotting the logarithm of the molecular mass of each standard against its Ve/V0 value. The apparent molecular mass of *Tc*LysRS^cyt^ was estimated from its elution volume and compared with the theoretical molecular mass of the full-length protomer, 66 kDa, to infer its oligomeric state in solution.

### 5.10 Enzyme Kinetics and Inhibition Assays

*Tc*LysRS^cyt^ enzymatic assays used an enzyme-coupled approach to examine the first step of aminoacylation, which involves the formation of an aminoacyl-adenylate intermediate with subsequent release of lysine, AMP, and pyrophosphate (PPi). In this system, the conversion of resazurin to resorufin served as the fluorescent marker for AMP formation. Reactions were prepared in black 384-well plates, with a final volume of 25 μL, and conducted at 25 °C using 0.5 μM of *Tc*LysRS^cyt^ in reaction buffer (50 mM Tris–HCl, pH 7.5, 0.15 M KCl, 0.15 M NaCl, 10 mM MgCl_2_, 0.01% Tween 20), containing 0.25 μM ScAMD1, 200 μM NAD^+^, 0.5 μM CjIMPDH, 25 μM resazurin, and 1 U·mL^–1^ *Clostridium kluyveri* Diaphorase (E.C. 1.6.99.3; Worthington Biochemical). Kinetic parameters were determined by varying lysine or ATP concentrations, keeping the other substrate at a constant, saturating level (10-fold excess). Resorufin fluorescence (λ_Exc_ = 545 – 20 nm; and λ_Em_ = 600 – 40 nm) was measured using a CLARIOStar microplate reader (BMG LabTech, Ortenberg, Germany). Fluorescence signals were converted to amounts of AMP formed using an AMP standard curve. The data were analyzed with GraphPad Prism v.10.

*Tc*LysRS^cyt^ inhibition assays were conducted using the same methodology described for the kinetic studies, adjusting the final concentrations of lysine and ATP to 1 mM and 500 μM, respectively. Positive controls (100% activity) were made without any inhibitor, but with the same amount of DMSO used in the test samples. Negative controls (0% activity) were made without l-Lys. Compound 5 was prepared in DMS, and 25 nL was transferred to assay plates using an acoustic liquid handler (Echo650, Beckman Coulter, Indianapolis, IN, USA). The data obtained were normalized based on controls (positive and negative), and the *IC*_50_ values were obtained by nonlinear regression of the data using GraphPad Prism v.10, according to the equation: In*h* = 100/(1 + 10^((log IC_50_-X)*Hill-slope)^), where Inh is the percentage of inhibition, log IC_50_ is the logarithm of the concentration that inhibits 50% of the response, X is the logarithm of the inhibitor concentration, and Hill-slope is the Hill coefficient. Each test was performed with four technical replicates and two biological replicates. The plot, calculation, and statistics were analyzed in GraphPad Prism v.10.

### 5.11 Differential Scanning Fluorimetry Assays

DSF was used to evaluate the effect of l-Lys and compound 5 on the thermal stability of TcLysRS^cyt^. The assay was carried out in 96-well plates in a final volume of 20 μL of buffer C containing purified *Tc*LysRS^cyt^ (5 μM) and SYPRO Orange (5*x*), in the absence or presence of compound 5 (50 μM) and/or l-Lys (5 mM). DMSO, used as the solvent for compound 5 stock solutions, was included as control. Protein unfolding was monitored on a CFX Real Time (Biorad, California, USA) cycler by measuring fluorescence during a temperature ramp from 25 to 95 °C with increments of 1 °C. Fluorescence data were normalized, and the negative first derivative (-dF/dT) was calculated. Melting temperatures (*T*_m_ values) were determined from the minimums of first derivative curves.

### 5.12 *Tc*LysRS^cyt^ Crystallization and Structure Determination

Following the purification of the recombinant full-length *Tc*LysRS^cyt^, the protein was concentrated to 7.1 mg·mL^–1^ in buffer C containing 50 mM TrisHCl (pH 8.0) and 300 mM NaCl. Crystallization experiments were performed at 18 °C utilizing the sitting-drop vapor-diffusion method. The screening plates were prepared with the Mosquito automated protein crystallization system (TTP Labtech, Melbourn, UK). Initial screening identified condition F02 from SG1 screen kit (Molecular Dimensions, Maumee, OH, USA) as a suitable starting point. The final optimized crystallization condition was established using the hanging-drop method and consisted of 1 μL of the protein solution (prepared as described above) mixed with 1 μL of a precipitant solution containing 0.1 M HEPES (pH 7.5), 0.4 M sodium acetate, and 22–28% PEG 3350. Crystals were grown either in the presence of AMP, lysine, and MgCl_2_ (*Tc*LysRS^cyt^-lysine complex) or with compound 5, lysine, and MgCl_2_ (*Tc*LysRS^cyt^-lysine-5 complex). Prior to X-ray diffraction data collection, crystals were cryoprotected with 15% PEG 400. Diffraction data sets were collected at 100 K using the Sirius MANACÁ Beamline at the Brazilian Synchrotron Light Laboratory (LNLS) at CNPEM.

Diffraction images obtained from *Tc*LysRS^cyt^ crystals were processed using XDS^37^ for indexing and integration. The resulting data were subsequently scaled with AIMLESS,^38^ as implemented in the CCP4i2 suite.^39^ The structures were determined by molecular replacement using PHASER,^40^ with the *P. falciparum* LysRS structure (PDB ID 6HCU) serving as the search model. Manual model building was performed using Coot.^41^ Additional refinement cycles are utilized either Refmac^42^ or PHENIX^43,44^ to improve the accuracy of the atomic model. Model geometry was carefully inspected throughout the process with Coot validation tools. The final refined structures were deposited in the Protein Data Bank under the accession codes 9YJN and 9YJR. Crystallographic statistics are available in the Supporting Information (Supporting Information appendix, Table S1). Figures illustrating the structures were prepared using the open-source PyMOL Molecular Graphics System, Version 2.5.0 (Schrödinger, LLC, New York, NY, USA).

## Supplementary Material





## Data Availability

Crystallographic structures have been deposited in the PDB under accession codes 9YJN and 9YJR. All other data substantiating the findings of this study are available in the manuscript or the Supporting Information (appendix). Additional raw data or materials that are not included in these sources are available from the corresponding authors upon reasonable request.

## Notes

Crystallographic data collection and refinement statistics; purity and homogeneity of recombinant *of Tc*LysRS^cyt^; differential scanning fluorimetry analysis; structural analysis of *Tc*LysRS^cyt^; and sequence alignment of cytosolic LysRS orthologs from human and parasitic organisms (PDF)
Molecular Formula Strings (CSV)

## ⊥

Renato Archer Center for Information Technology, 13069-901, Campinas, SP, Brazil

## Abbreviations Used

*Cj*IMPDH: *Campylobacter jejuni* inosine monophosphate dehydrogenase
*Cp*LysRS: *Cryptosporidium parvum* lysyl-tRNA synthetase
DSF: differential scanning fluorimetry
HsLysRS: human lysyl-tRNA synthetase
LMN: lymphomononuclear infiltrate
LMNP: lymphomononuclear infiltrate with polymorphonuclear neutrophils
LysRS: lysyl-tRNA synthetase
NCA: noncompartmental analysis
*Pf*LysRS: *Plasmodium falciparum* lysyl-tRNA synthetase
qPCR: quantitative polymerase chain reaction
*Sc*AMD1: *Saccharomyces cerevisiae* adenosine monophosphate deaminase
SEC: size-exclusion chromatography
*Tc*LysRS^cyt^: cytosolic *Trypanosoma cruzi* lysyl-tRNA synthetase
UTI: untreated infected
UUI: untreated uninfected
