# Supplementary material for: In Vivo Efficacy and Biochemical Target Engagement of a Chromene-Based Inhibitor of Trypanosoma cruzi Lysyl-tRNA Synthetase
Source: J Med Chem. 2026 Aug 13;69(17):20313–25. doi: 10.1021/acs.jmedchem.6c00764 (PMC13576447; doi:10.1021/acs.jmedchem.6c00764)
Supplement: Supplementary file 1 [file jm6c00764_si_001.pdf]

Supporting Information for

In Vivo Efficacy and Biochemical Target Engagement of a Chromene-based Inhibitor of Trypanosoma cruzi Lysyl-tRNA Synthetase

Thaís Cristina Ferreira dos Santos[1, †], Caio Cesar de Lima Silva[1, †, ‡], Amanda Gonçalves Eufrásio[1,2], Cristiane Tambascia[1], Irene Layane de Sousa[1], Michelle Fagundes Catelli[1], Letícia Marchese[1], Leonardo Rodrigues de Almeida[3], Giovana da Costa Venancio[1], Valéria Barbosa de Souza[4], André Almeida Schenka[4], Silvana Aparecida Rocco[1], Gustavo Fernando Mercaldi[1,*], Artur Torres Cordeiro[1,*]

[1]Brazilian Biosciences National Laboratory (LNBio), Brazilian Center for Research in Energy and Materials (CNPEM), 13083-100, Campinas – SP, Brazil
[2]School of Pharmaceutical Sciences, Universidade Estadual de Campinas (UNICAMP), 13083-871, Campinas - SP, Brazil
[3]Theoretical and Structural Chemistry Group, Universidade Estadual de Goiás, Anápolis, GO, Brazil
[4]Department of Pharmacology, School of Medical Sciences, Universidade Estadual de Campinas (UNICAMP), 13.083-888, Campinas - SP, Brazil

[†] These authors contributed equally to this work.
[‡] Present address: Renato Archer Center for Information Technology, 13069-901, Campinas - SP, Brazil.

Corresponding authors

Artur Torres Cordeiro, artur.cordeiro@lnbio.cnpem.br. Brazilian Biosciences National Laboratory (LNBio), Brazilian Center for Research in Energy and Materials (CNPEM), Rua Giuseppe Maximo Scolfaro 10,000, Campinas - SP, 13083-100, Brazil.

Gustavo Fernando Mercaldi, gustavo.mercaldi@lnbio.cnpem.br. Brazilian Biosciences National Laboratory (LNBio), Brazilian Center for Research in Energy and Materials (CNPEM), Rua Giuseppe Maximo Scolfaro 10,000, Campinas - SP, 13083-100, Brazil.

This PDF includes:

Table S1. Crystallographic data collection and refinement statistics.
Figure S1. Purity and homogeneity of recombinant cytosolic lysyl-tRNA synthetase from *Trypanosoma cruzi* (*Tc*LysRS^{cyt}).
Figure S2. Differential scanning fluorimetry analysis of *Tc*LysRS^{cyt} in the absence and presence of ligands.
Figure S3. Structure of *Trypanosoma cruzi* lysyl-tRNA synthetase (*Tc*LysRS^{cyt}).
Figure S4. Sequence alignment of cytosolic lysyl-tRNA synthetases (LysRS) from human and parasitic organisms.

Table S1. Crystallographic data collection and refinement statistics.

	*Tc*KRSc-Lys	*Tc*KRSc-5-Lys
**Wavelength**	0.9772	0.9772
**Resolution range**	47.45 - 2.08 (2.13 - 2.08)	47.33 - 2.8 (3.02 - 2.8)
**Space group**	P 43 21 2	P 43 21 2
**Unit cell (x, y, z)**	59.97, 59.97, 310.3	60.1 60.1 307.177
**(α, β, γ)**	90, 90, 90	90 90 90
**Total reflections**	893993 (65717)	205109 (35324)
**Unique reflections**	35668 (2646)	16747 (3243)
**Multiplicity**	25.1 (24.8)	12.2 (10.9)
**Completeness (%)**	99.8 (97.5)	96.87 (84.44)
**Mean I/sigma(I)**	14.3 (1.5)	5.99 (0.85)
**Wilson B-factor**	47.8	95.4
**R-merge**	0.156 (2.999)	0.2915 (2.998)
**R-meas**	0.159 (3.061)	0.3041 (3.145)
**R-pim**	0.043 (0.842)	0.08589 (0.9403)
**CC1/2**	0.998 (0.456)	0.996 (0.398)
**Reflections used in refinement**	33768	14393
**Free reflections (%)**	5	5
**R-work**	0.19	0.26
**R-free**	0.25	0.31
**Number of non-hydrogen atoms**	4031	3594
**macromolecules**	3955	3566
**ligands**	10	25
**solvent**	66	3
**Protein residues**	461	490
**RMS(bonds)**	0.93	2
**RMS(angles)**	1.41	0.44
**Ramachandran**		
**favored (%)**	93	94
**allowed (%)**	6	6
**outliers (%)**	0.6	0.2
**Rotamer outliers (%)**	3.73	1.60
**Clashscore**	7.74	3.87
**Average B-factor (all atoms)**	63.00	117.47
**macromolecules**	63.75	117.70
**ligands**	45	90.55
**solvent**	48.9	70.58
**PDB ID**	9YJN	9YJR

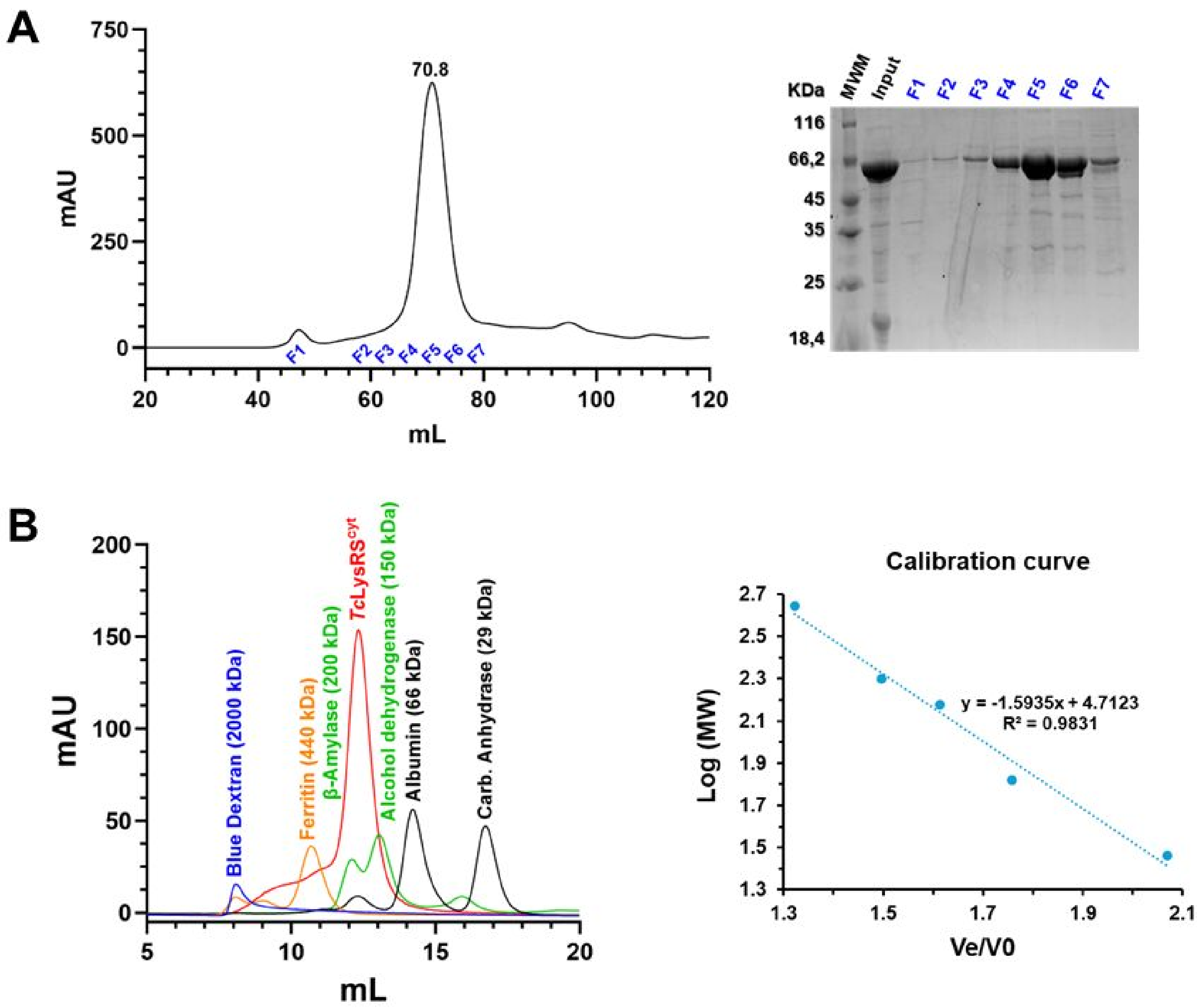

Figure S1. Purity and homogeneity of recombinant cytosolic lysyl-tRNA synthetase from *Trypanosoma cruzi* (*Tc*LysRS^{cyt}). A) Representative preparative size-exclusion chromatography (SEC) profile and SDS-PAGE analysis of *Tc*LysRS^{cyt} samples obtained during the final purification steps. **B)** Analytical SEC analysis showing that full-length *Tc*LysRS^{cyt} (red line) eluted as a predominant oligomeric species with an apparent molecular weight of approximately 190 ± 20 kDa. This apparent mass exceeds the theoretical dimeric mass (~132 kDa), likely reflecting the hydrodynamic behavior of the protein in SEC, but supports that *Tc*LysRS^{cyt} is not monomeric in solution. Molecular weight standards are indicated in the chromatogram; each line represents an independent run.

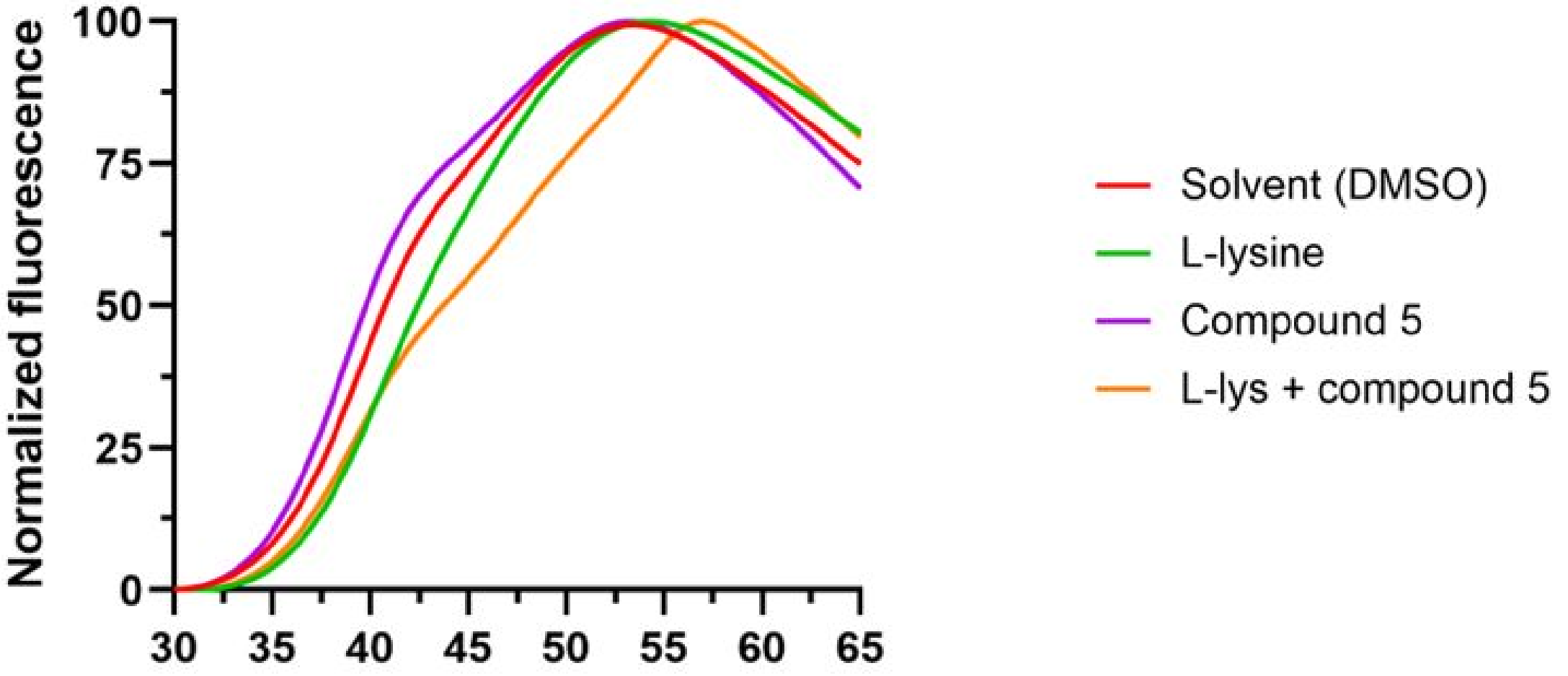

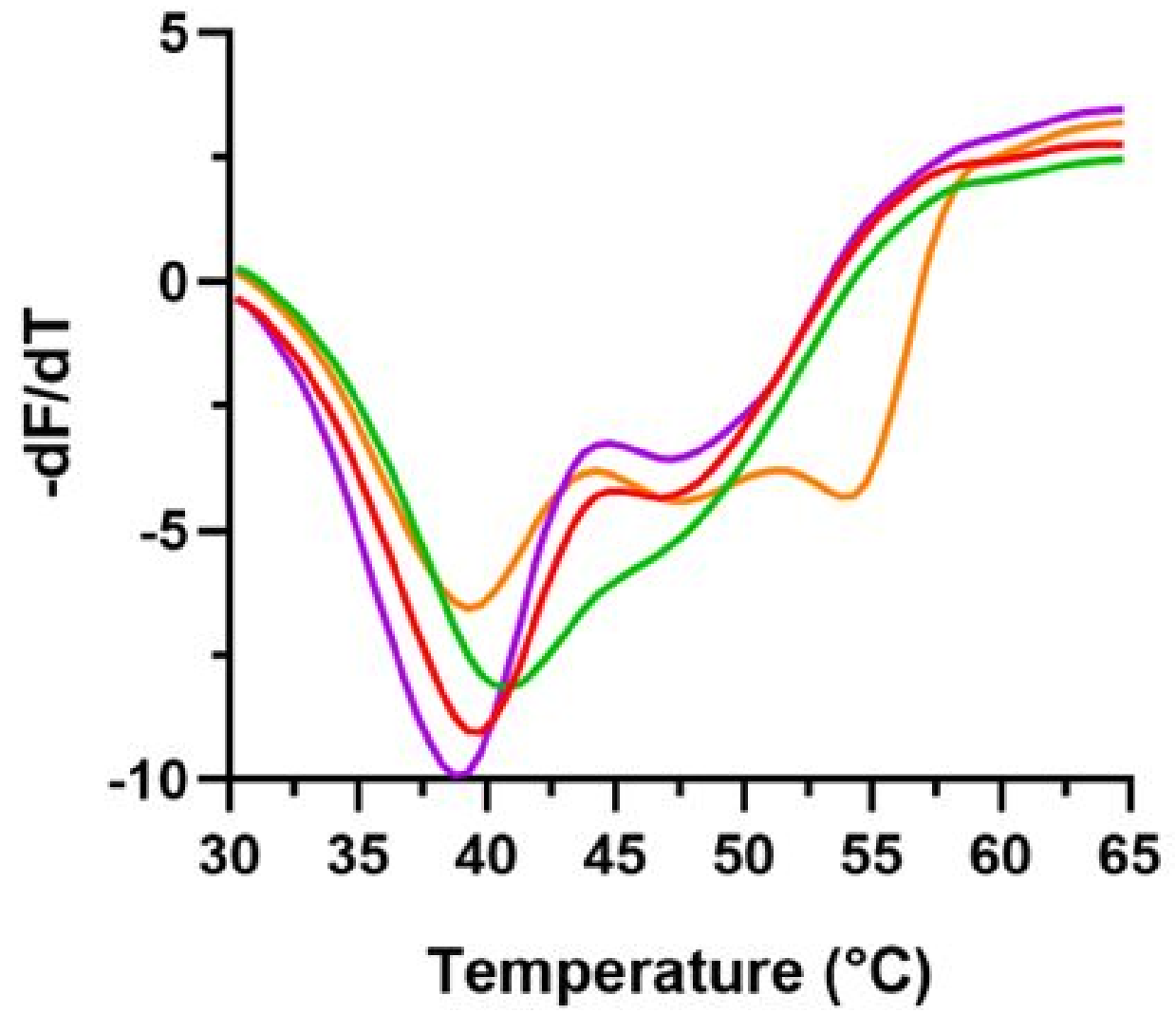

Ligand	Tm¹	Tm²	Tm³
**Solvent**	39.8	46.8	-
**L-lysine**	40.8	-	-
**Compound 5**	38.8	47.3	-
**L-lysine + compound5**	39.3	47.3	53.8

Figure S2. Differential scanning fluorimetry analysis of *Tc*LysRS^{cyt} in the absence and presence of ligands. Thermal shift denaturation curves show that *Tc*LysRS^{cyt} displays a biphasic unfolding profile both in the apo condition and in the presence of individual ligands, such as lysine or compound 5. In contrast, the combined presence of lysine and compound 5 produces an additional transition phase, suggesting that simultaneous ligand binding stabilizes a distinct conformational or population state of the enzyme.

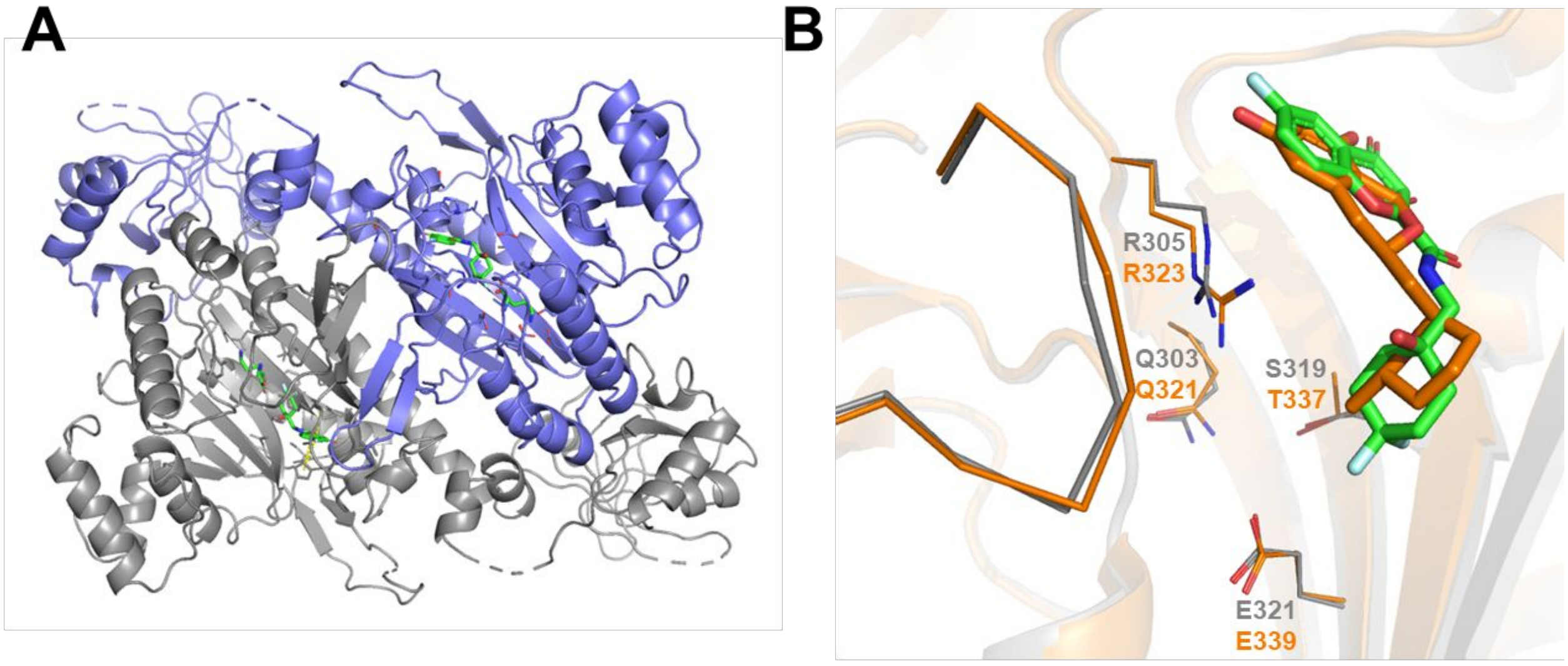

Figure S3. Structure of *Trypanosoma cruzi* lysyl-tRNA synthetase (*Tc*LysRS^{cyt}). A) Overall *Tc*LysRS structure and visualization of the dimer (with two protomers, one depicted in blue and the other as gray), reconstituted through crystallographic symmetry; the active site contains both lysine and compound 5, illustrated as green sticks. **B)** Structural alignment of *Tc*LysRS (gray) and human LysRS (*Hs*LysRS; orange), each complexed with compound 5 (sticks with green carbons) and cladosporin (sticks with orange carbons), respectively, demonstrate key residues involved in stabilizing an adjacent loop. These residues are conserved between human and *T. cruzi* LysRS, but differ in *Plasmodium* and *C. parvum* LysRS, which has importance for compound selectivity.

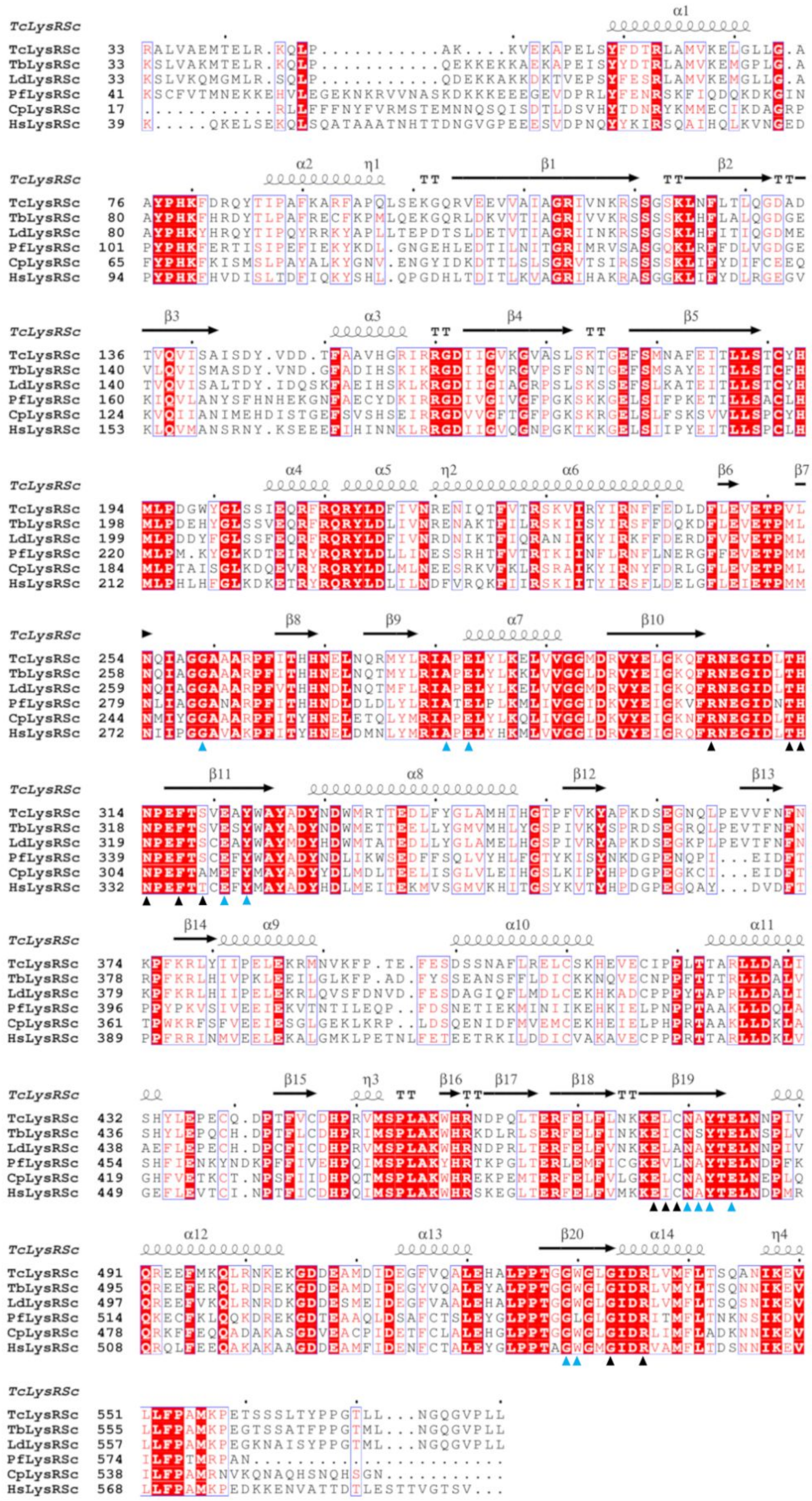

Figure S4. Sequence alignment of cytosolic lysyl-tRNA synthetases (LysRS) from human and parasitic organisms. Residues forming the ATP- (black arrowheads) and L-lysine-binding pockets (blue arrowheads) are indicated. Compound 5 is an inhibitor that binds to the ATP pocket. Secondary-structure elements of *Tc*LysRS^{cyt} are shown above the aligned sequences. The sequence alignment was generated using Clustal Omega[1] and rendered with ESPript 3.2.[2] Abbreviations: **Tc**, *T. cruzi*, **Tb**, *T. brucei*, **Ld**, *L. donovani*, **Pf**, *P. falciparum*, **Cp**, *C. parvum*, **Hs**, *H. sapiens.*

Supplementary References

(1) Madeira, F.; Madhusoodanan, N.; Lee, J.; et al. The EMBL-EBI Job Dispatcher sequence analysis tools framework in 2024. *Nucleic Acids Research*. **2024,** 52(W1):W521-W525. DOI: 10.1093/nar/gkae241.

(2) Gouet, P.; Robert, X.; and Courcelle, E. ESPript/ENDscript: extracting and rendering sequence and 3D information from atomic structures of proteins. *Nucl. Acids Res*. **2003**, 31(13), 3320-3323. doi: 10.1093/nar/gkg556.
